# Machine Learning–Based Selection of Resection vs Transplant and Survival in Hepatocellular Carcinoma

**DOI:** 10.1001/jamanetworkopen.2025.32353

**Published:** 2025-09-17

**Authors:** Hyun Uk Kim, Ji Won Han, Pil Soo Sung, Jeong Won Jang, Seung Kew Yoon, Ho Joong Choi, Young Kyoung You

**Affiliations:** 1 College of Medicine, The Catholic University of Korea, Seoul, Republic of Korea; 2 The Catholic University Liver Research Center, College of Medicine, The Catholic University of Korea, Republic of Korea; 3 Division of Gastroenterology and Hepatology, Department of Internal Medicine, Seoul St Mary’s Hospital, College of Medicine, The Catholic University of Korea, Seoul, Republic of Korea; 4 Department of Surgery, Seoul St Mary’s Hospital, College of Medicine, The Catholic University of Korea, Seoul, Republic of Korea

## Abstract

**Importance:**

Liver transplantation (LT) generally provides superior long-term survival compared with surgical resection (SR) for hepatocellular carcinoma (HCC), but optimal treatment selection remains challenging due to donor scarcity and patient heterogeneity.

**Objective:**

To develop and validate a machine learning (ML)–based decision-support model to estimate optimized individualized treatment selection between LT and SR in HCC.

**Design, Setting, and Participants:**

This nationwide cohort study included patients with HCC who underwent either LT or SR between 2008 and 2018 from the Korea Central Cancer Registry as the derivation cohort. An independent cohort of patients with HCC treated between 2009 and 2020 at Seoul St Mary’s Hospital was used for external validation. Data were analyzed from February to March 2025.

**Exposures:**

Curative treatment with LT or SR for HCC.

**Main Outcomes and Measures:**

Separate ML models estimating 3-year overall survival (OS) were developed for LT and SR. Patients were stratified into high- and low-risk groups for each treatment, identifying LT-favorable and LT-nonfavorable groups. Counterfactual analysis evaluated OS differences between ML-guided and clinical practice treatments.

**Results:**

A total of 3915 patients (3137 [80.1%] male), 296 in the LT group (median [IQR] age, 54.0 [49.0-60.0] years) and 3619 in the SR group (median [IQR] age, 58.0 [51.0-66.0] years), were included in the derivation cohort, and 614 patients (497 [80.9%] male)—314 in the LT group (median [IQR] age, 55.0 [51.0-60.0] years) and 300 in the SR group (median [IQR] age, 59.0 [52.0-66.0] years)—were included in the external validation cohort. Across both cohorts, LT recipients were generally younger and had more advanced liver disease, with higher rates of cirrhosis (78 [26.4%] vs 699 [19.3%]; *P* = .005), hepatic encephalopathy (20 [6.8%] vs 10 [0.3%]; *P* < .001), and ascites (50 [19.9%] vs 153 [4.2%]; *P* < .001). LT recipients also exhibited poorer liver function, with lower albumin levels (median [IQR], 3.4 [2.8-4.0] vs 4.2 [3.9-4.5] g/dL), higher bilirubin levels (median [IQR], 1.4 [0.9-2.5] vs 0.7 [0.5-1.0] mg/dL), and prolonged international normalized ratios (median [IQR], 1.2 [1.1-1.5] vs 1.1 [1.0-1.1]), and had smaller tumors (median [IQR], 2.3 [1.5-3.6] vs 3.2 [2.2-5.0] cm; *P* < .001) but more tumors (mean [SD], 1.6 [1.0] vs 1.2 [0.7]; *P* < .001). The support vector machine model achieved the highest area under the receiver operating characteristic curve (AUROC) of 0.82 (95% CI, 0.78-0.86) in the LT cohort, whereas CatBoost performed best in the SR cohort (AUROC, 0.79 [95% CI, 0.78-0.80]). Counterfactual analysis estimated that ML-guided treatment decisions could improve survival compared with observed clinical practice decisions (HR, 0.46 [95% CI, 0.42-0.50]; *P* < .001). These findings were consistent in the independent cohort.

**Conclusions and Relevance:**

Findings from this cohort study of patients with HCC indicated that an ML-based decision-support model estimated accurate risk stratification and identified the potential for improved survival through individualized, model-guided treatment selection. These findings suggest clinical utility in supplementing existing guidelines.

## Key Points

**Question:**

Can machine learning (ML)–based risk stratification be used to optimize individualized treatment selection between liver transplantation (LT) and surgical resection for patients with hepatocellular carcinoma (HCC)?

**Findings:**

In this cohort study of 3915 patients with HCC, ML models stratified treatment-specific risk and identified an LT-favorable group. Counterfactual analysis suggested that ML-guided decisions may potentially improve survival compared with clinical practice decisions, with consistent findings in an external validation cohort.

**Meaning:**

Findings from this study suggested that ML-based decision-support models can stratify patients with HCC by treatment-related risk, enabling personalized, risk-aligned treatment selection between LT and surgical resection and potentially improving survival outcomes through optimized clinical decision-making.

## Teaser

This cohort study develops and validates a machine learning–based decision-support model to assess whether it can be used for risk stratification to optimize individualized treatment selection between liver transplantation and surgical resection among patients with hepatocellular carcinoma.

## Introduction

Hepatocellular carcinoma (HCC) remains a leading cause of cancer-related mortality worldwide. For early-stage cases, typically Barcelona Clinic Liver Cancer stage 0 or A, liver transplantation (LT) and surgical resection (SR) are the primary curative options.^1,2^ Several studies have demonstrated that short-term overall survival (OS) is comparable between SR and LT (1-year OS, 84.5% vs 84.4%; 3-year OS, 65.3% vs 69.6%, respectively).^3,4^ However, LT consistently provides superior long-term outcomes, with 5-year OS and disease-free survival rates of 59.3% and 62.5%, respectively, compared with 47.9% and 35.6%, respectively, for SR. This survival advantage persists at 10 years, with LT achieving 50.0% OS and 45.2% disease-free survival, whereas SR decreases to 29.8% and 18.1%, respectively.^4,5^

Treatment selection between SR and LT is primarily determined by liver function, tumor burden, and the extent of underlying cirrhosis. SR is preferred for patients with preserved hepatic function (Child-Pugh A, no significant portal hypertension) and limited tumor burden, typically a solitary lesion without major vascular invasion.^1,6^ In contrast, LT is indicated for patients meeting the Milan criteria.^1,6^ Despite its survival benefits, LT is constrained by donor shortages, leading to long wait times and risk of disease progression.^7^ Additionally, lifelong immunosuppression poses risks of infection, kidney dysfunction, metabolic complications, and secondary malignant tumors.^8^

Recent studies have focused on identifying prognostic factors and developing prediction models to estimate survival and recurrence outcomes following LT and SR for HCC. Prognosis after curative treatment is influenced by tumor size, number, vascular invasion, differentiation, liver function, and viral hepatitis status.^9,10^ Several prognostic models have been developed incorporating tumor burden, biological markers, such as alpha-fetoprotein (AFP) and protein induced by vitamin K absence-II (PIVKA-II), and liver function parameters, including bilirubin and Model for End-Stage Liver Disease score.^11,12^ Incorporation of imaging features into these models has further enhanced predictive accuracy and risk stratification.^13,14^ Emerging machine learning (ML) models, such as random survival forests and neural networks, demonstrate improved predictive performance over traditional methods by modeling complex, nonlinear interactions.^15,16,17,18^

While ML-based prognostic models have demonstrated promise in predicting survival and recurrence after each treatment,^15,16,17,18^ no ML-based tool currently guides treatment selection between SR and LT. Given the distinct risks and benefits of each approach, an individualized, data-driven strategy may optimize patient outcomes. This study aimed to develop and validate an ML-based decision-support model to inform treatment selection between SR and LT for patients with HCC.

## Methods

### Study Patients

In this cohort study, data from the nationwide Korea Central Cancer Registry were used as a derivation cohort, including all patients with HCC who underwent LT or SR between 2008 and 2018. An independent external validation cohort was retrospectively obtained from Seoul St Mary’s Hospital, including patients with HCC who underwent LT or SR between 2009 and 2020. Patients lost to follow-up within 3 years were excluded. The study was approved by the institutional review board of the Catholic University of Korea, which also waived the need for informed consent due to the retrospective nature of this study. This study was conducted following the Strengthening the Reporting of Observational Studies in Epidemiology (STROBE) reporting guideline.

### ML-Based Risk Modeling

The derivation cohort was split into a 7:3 ratio for training and testing while maintaining class distribution. Separate ML models were developed to estimate 3-year OS for LT and SR. Six ML classifiers were implemented: logistic regression, random forest, support vector machine (SVM), XGBoost, LightGBM, and CatBoost.^19,20,21^ A total of 30 variables, including demographic factors, clinical characteristics, and tumor-related variables, were used for analysis (eTable 1 in Supplement 1). All variables were collected at the most recent time point prior to treatment initiation. Missing numerical data were imputed using Iterative Imputer,^22^ while missing categorical values were imputed using probabilistic sampling, as detailed in the eMethods in Supplement 1. Model performance was evaluated using accuracy, precision, recall, F1 score, and the area under the receiver operating characteristic curve (AUROC), with 95% CIs estimated via 1000 bootstrap resamples. Patients were stratified into high- and low-risk groups based on an optimal threshold that maximized the F1 score. To enhance interpretability, Shapley additive explanation (SHAP) analysis was conducted.

### Treatment Selection Modeling

Using the selected ML models, we estimated each patient’s 3-year mortality risk for both LT and SR. The ML-recommended treatment was defined as the option yielding the lower estimated mortality risk. Based on these estimations, patients were categorized into LT-favorable or LT-nonfavorable groups. To evaluate the potential benefit of ML-guided treatment selection, a counterfactual analysis was conducted using a Cox proportional hazards model.^23^ For each patient, a survival curve under the ML-recommended treatment was estimated using the estimated risk as a linear predictor and compared with the observed survival under the actual treatment received. Additional details of the method are provided in the eMethods in Supplement 1.

### Statistical Analysis

Data were analyzed from February to March 2025. Continuous variables are summarized as medians with IQRs, reported as the 25th percentile to the 75th percentile, and compared using the Student *t* test or Mann-Whitney *U* test, as appropriate. Categorical variables are presented as numbers and percentages and were analyzed using either the χ^2^ test or Fisher exact test. Survival outcomes were compared using Kaplan-Meier analysis and the log-rank test. Statistical significance was considered to be a 2-sided *P* < .05. This study was conducted using Python, version 3.12.2 (Python Software Foundation), scikit-learn, version 1.5.2, XGBoost, version 2.1.3, LightGBM, version 4.5.0, CatBoost version, 1.2.7, and R, version 4.3.3 (R Project for Statistical Computing).

## Results

### Patient Characteristics

The derivation cohort included 3915 patients (778 [19.9%] female and 3137 [80.1%] male), 3619 who underwent SR and 296 who underwent LT. The baseline characteristics of the derivation cohort are summarized in eTable 1 in Supplement 1. Compared with SR recipients, LT recipients were younger (median [IQR] age, 54.0 [49.0-60.0] vs 58.0 [51.0-66.0] years; *P* < .001) and exhibited a higher prevalence of cirrhosis (78 [26.4%] vs 699 [19.3%]; *P* = .005), hepatic encephalopathy (20 [6.8%] vs 10 [0.3%]; *P* < .001), and ascites (50 [19.9%] vs 153 [4.2%]; *P* < .001). They also had poorer liver function, with lower albumin levels (median [IQR], 3.4 [2.8-4.0] vs 4.2 [3.9-4.5] g/dL; to convert to grams per liter, multiply by 10), higher bilirubin levels (median [IQR], 1.4 [0.9-2.5] vs 0.7 [0.5-1.0] mg/dL; to convert to micromoles per liter, multiply by 17.104), prolonged international normalized ratio (INR) (median [IQR], 1.2 [1.1-1.5] vs 1.1 [1.0-1.1]), lower sodium levels (median [IQR], 139.0 [136.0-141.0] vs 140.0 [138.0-142.0] mEq/L; to convert to millimoles per liter, multiply by 1.0), and reduced platelet counts (median [IQR], 87.0 [59.5-126.5] vs 167.0 [130.0-212.0] × 10^3^/µL; to convert to 10^9^ per liter, multiply by 1.0) (all *P* < .001). Regarding tumor-related factors, the LT group had smaller maximum tumor sizes (median [IQR], 2.3 [1.5-3.6] vs 3.2 [2.2-5.0] cm; *P* < .001) but a higher number of tumors (mean [SD], 1.6 [1.0] vs 1.2 [0.7]; *P* < .001). The external validation cohort had characteristics similar to the derivation cohort (eTable 2 in Supplement 1).

### Development, Selection, and Interpretation of ML-Based Survival Models

For the LT cohort, the support vector machine model achieved the best performance (AUROC, 0.82 [95% CI, 0.78-0.86]; F1 score, 0.60 [95% CI, 0.54-0.66]). For the SR cohort, CatBoost performed best (AUROC, 0.79 [95% CI, 0.78-0.80]; F1 score, 0.51 [95% CI, 0.49-0.53]). Complete model performance metrics are detailed in eTable 3 in Supplement 1. SHAP analysis identified key factors to model outcomes. In the LT cohort, tumor number, platelet count, and PIVKA-II, creatinine, AFP, and total bilirubin levels were key factors. In the SR cohort, maximum tumor size, INR, and AFP, albumin, PIVKA-II, and sodium levels were key factors (eFigure 1 in Supplement 1).

### Survival Outcomes Following ML-Based Risk Stratification

**Figure 1.**
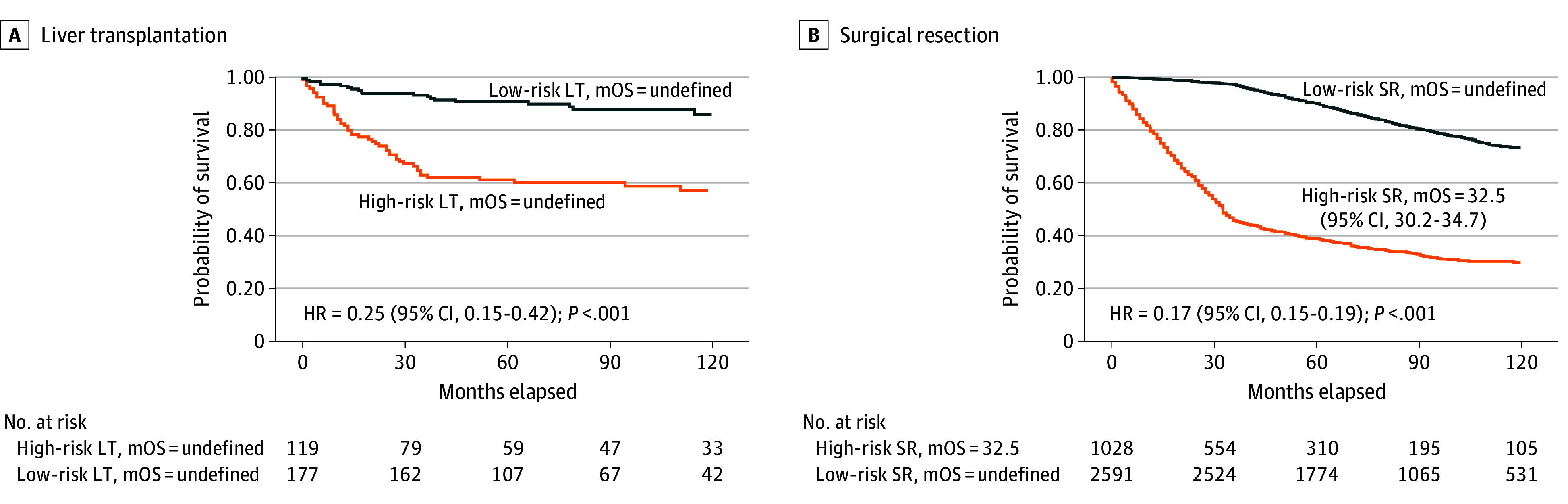
Kaplan-Meier Survival Analysis of Machine Learning–Defined Risk Groups in the Derivation Cohort Patients were stratified into high- and low-risk groups using a support vector machine model for liver transplantation (LT) and a CatBoost model for surgical resection (SR). A, Kaplan-Meier survival curves comparing high-risk (n = 119) and low-risk (n = 177) groups within the LT cohort (n = 296). B, Kaplan-Meier survival curves comparing high-risk (n = 1028) and low-risk (n = 2591) groups within the SR cohort (n = 3619). HR indicates hazard ratio; mOS, median overall survival.

ML-based risk stratification resulted in 119 patients categorized as high risk and 177 patients categorized as low risk in the LT cohort. Kaplan-Meier analysis showed significantly better survival in the low-risk group (hazard ratio [HR], 0.25 [95% CI, 0.15-0.42]; *P* < .001) (Figure 1A). Median overall survival (mOS) was not reached in either group. In the SR cohort, 1028 patients were categorized as high risk, and 2591 patients as low risk. The low-risk group exhibited significantly better survival (HR, 0.17 [95% CI, 0.15-0.19]; *P* < .001) (Figure 1B). The mOS was 32.5 months (95% CI, 30.2-34.7 months) in the high-risk group and was not reached in the low-risk group.

**Figure 2.**
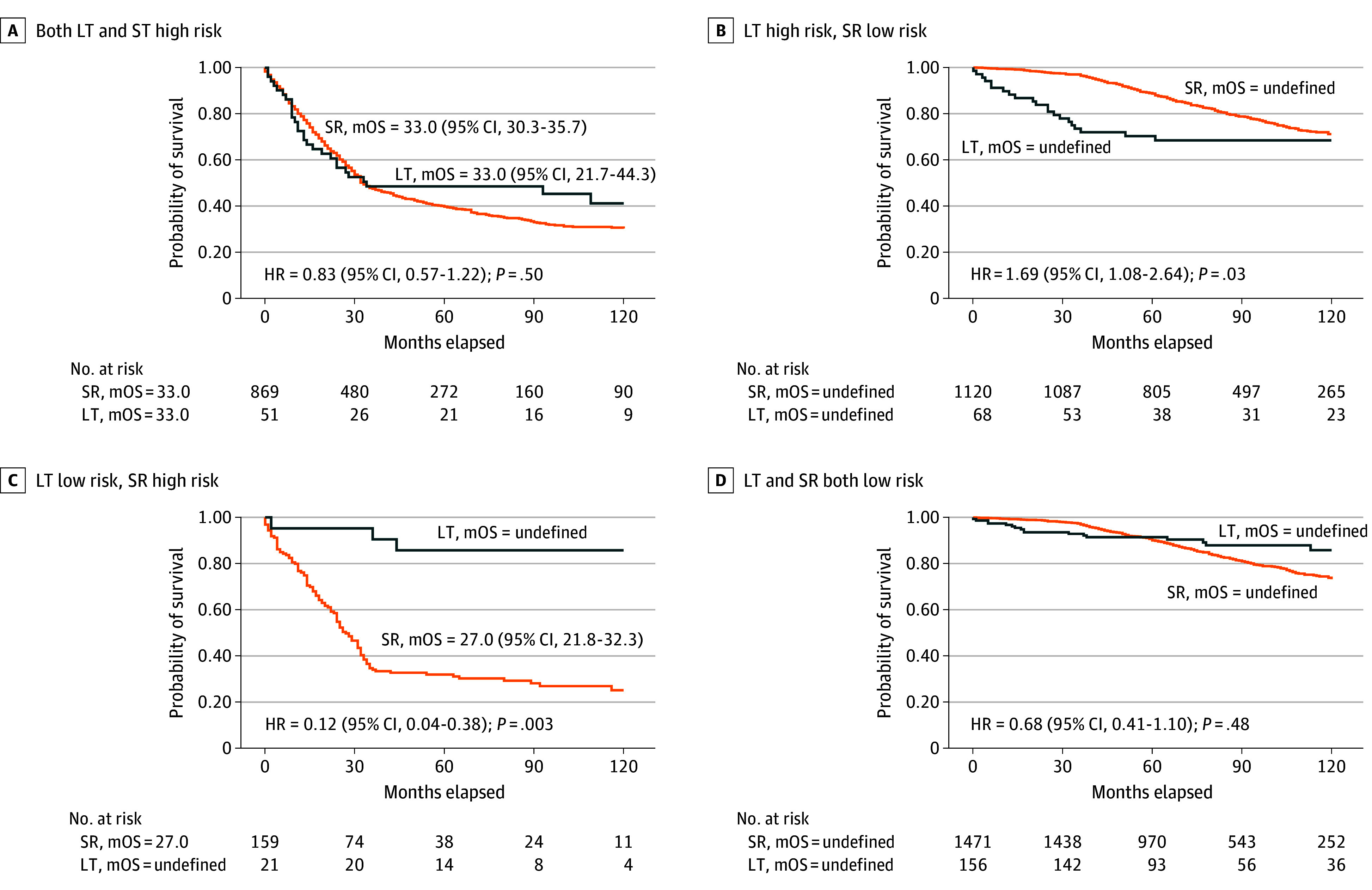
Combinatorial Machine Learning (ML)–Based Risk Stratification and Survival Analysis in the Derivation Cohort Patients (n = 3915) were classified into 4 risk groups based on ML-predicted survival probabilities for liver transplantation (LT) and surgical resection (SR). Kaplan-Meier survival curves comparing LT (n = 51) and SR (n = 869) in the group with both LT and SR high risk (n = 920) (A); LT (n = 68) and SR (n = 1120) in the LT high-risk, SR low-risk group (n = 1188) (B); LT (n = 21) and SR (n = 159) in the LT low-risk, SR high-risk group (n = 180) (C); and LT (n = 156) and SR (n = 1471) in the group with LT and SR both low risk (n = 1627) (D). HR indicates hazard ratio; mOS, median overall survival.

Patients were then categorized into 4 groups based on their estimated risk for LT and SR: (1) both LT and SR high risk (920 of 3915 [23.5%]), (2) LT high risk with SR low risk (1188 of 3915 [30.3%]), (3) LT low risk with SR high risk (180 of 3915 [4.6%]), and (4) both LT and SR low risk (1627 of 3915 [41.6%]). In the LT high with SR low group, LT was associated with worse survival compared with SR (HR, 1.69 [95% CI, 1.08-2.64]; *P* = .03). Conversely, in the LT low with SR high group, LT demonstrated a significant survival advantage over SR (HR, 0.12 [95% CI, 0.04-0.38]; *P* = .003) (Figure 2). These findings suggested that stratifying patients by estimated risk for LT and SR may help identify subgroups in which one treatment may offer a survival advantage over the other.

### Identification of Patients as LT-Favorable or LT-Nonfavorable

Patients were classified as LT-favorable or LT-nonfavorable according to which treatment—LT or SR—was associated with a lower estimated 3-year mortality risk. Among 296 LT recipients, the model recommended SR for 221 (74.7%). Among 3619 SR recipients, the model recommended LT for 701 (19.4%). Baseline characteristics for the LT-favorable (n = 776) and LT-nonfavorable (n = 3139) groups are presented in Table. Compared with the LT-nonfavorable group, the LT-favorable group was composed of patients who were older (median [IQR] age, 60.0 [53.0-68.0] vs 57.0 [51.0-65.0] years; *P* < .001). The LT-favorable group showed higher prevalence of ascites (62 [8.0%] vs 141 [4.5]%; *P* < .001) and hepatic encephalopathy (11 [1.4%] vs 19 [0.6%]; *P* = .04). Laboratory markers reflected poorer liver function, with lower albumin levels (median [IQR], 3.9 [3.5-4.2] vs 4.2 [3.9-4.5] g/dL), and higher bilirubin levels (median [IQR], 0.8 [0.6-1.2] vs 0.7 [0.5-1.0] mg/dL) and INR (median [IQR], 1.1 [1.0-1.2] vs 1.0 [1.0-1.1]) (all *P* < .001). Tumor markers were also elevated, including AFP (median [IQR], 42.1 [9.0-247.7] vs 11.3 [3.9-190.1] ng/mL) and PIVKA-II (median [IQR], 174.0 [45.7-942.7] vs 54.0 [25.0-329.0] mAU/mL) levels (both *P* < .001). Tumor burden was greater, with more tumors (mean [SD], 1.6 [1.2] vs 1.3 [0.8]) and larger tumor size (median [IQR], 4.0 [2.8-6.0] vs 3.0 [2.0-4.5] cm) (both *P* < .001).

**Table.** Comparison of Baseline Characteristics of the LT-Favorable and LT-Nonfavorable Groups

Characteristic	Patients, No. (%)		*P* value
	LT favorable (n = 776)	LT nonfavorable (n = 3139)	
Age, median (IQR), y	60.0 (53.0-68.0)	57.0 (51.0-65.0)	<.001
Sex			
Female	164 (21.1)	614 (19.6)	.35
Male	612 (78.9)	2525 (80.4)	
Height, median (IQR), cm	165.6 (159.7-170.6)	166.7 (160.7-171.0)	.02
Weight, median (IQR), kg	65.2 (57.8-72.8)	66.1 (59.5-73.5)	.02
Smoking, median (IQR), pack-years	0.0 (0.0-19.2)	0.0 (0.0-20.0)	.65
Diabetes	201 (25.9)	691 (22.0)	.02
Hypertension	286 (36.9)	1111 (35.4)	.47
Total cholesterol, median (IQR), mg/dL	153.0 (129.0-179.0)	162.0 (140.0-187.0)	<.001
HBV	492 (63.4)	2237 (71.3)	<.001
HCV	89 (11.5)	181 (5.8)	<.001
Alcohol	244 (31.4)	882 (28.1)	.07
Cirrhosis	163 (21.0)	614 (19.6)	.39
Encephalopathy	11 (1.4)	19 (0.6)	.04
Ascites	62 (8.0)	141 (4.5)	<.001
ECOG			
0	457 (58.9)	2135 (68.0)	<.001
1	288 (37.2)	286 (30.9)	
≥2	31 (3.9)	44 (1.1)	
Albumin, median (IQR), g/dL	3.9 (3.5-4.2)	4.2 (3.9-4.5)	<.001
Bilirubin, median (IQR), mg/dL	0.8 (0.6-1.2)	0.7 (0.5-1.0)	<.001
INR	1.1 (1.0-1.2)	1.0 (1.0-1.1)	<.001
Creatinine, median (IQR), mg/dL	0.8 (0.7-0.9)	0.9 (0.8-1.0)	<.001
Sodium, median (IQR), mEq/L	139.0 (137.0-141.0)	140.0 (138.0-142.0)	<.001
ALT, median (IQR), U/L	32.0 (22.0-52.0)	31.0 (22.0-47.0)	.01
Platelet count, median (IQR), 10^3^/µL	154.0 (108.0-202.0)	165.0 (126.0-210.0)	<.001
AFP, median (IQR), ng/mL	42.1 (9.0-247.7)	11.3 (3.9-190.1)	<.001
PIVKA-II, median (IQR), mAU/mL	174.0 (45.7-942.7)	54.0 (25.0-329.0)	<.001
Tumor burden, median (IQR)			
No.	1.0 (1.0-1.0)	1.0 (1.0-1.0)	<.001
Maximum size, cm	4.0 (2.8-6.0)	3.0 (2.0-4.5)	<.001
Invasion			
Portal vein	107 (13.8)	128 (4.1)	<.001
Hepatic vein	18 (2.3)	31 (1.0)	.005
Bile duct	12 (1.5)	37 (1.2)	.52

Abbreviations: AFP, alpha-fetoprotein; ALT, alanine aminotransferase; ECOG, Eastern Cooperative Oncology Group; HBV, hepatitis B virus; HCV, hepatitis C virus; INR, international normalized ratio; LT, liver transplantation; PIVKA-II, protein induced by vitamin K antagonist-II.

SI conversion factor: To convert albumin level to grams per liter, multiply by 10; ALT levels to microkatal per liter, by 0.0167; bilirubin level to micromoles per liter, by 17.104; total cholesterol to millimoles per liter, multiply by 0.0259; creatinine level to micromoles per liter, by 88.4; platelet count to 10^9^ per liter, by 1.0; and sodium level to millimoles per liter, by 1.0.

### Counterfactual Analysis and Survival Benefits of ML-Guided Treatment Decisions

**Figure 3.**
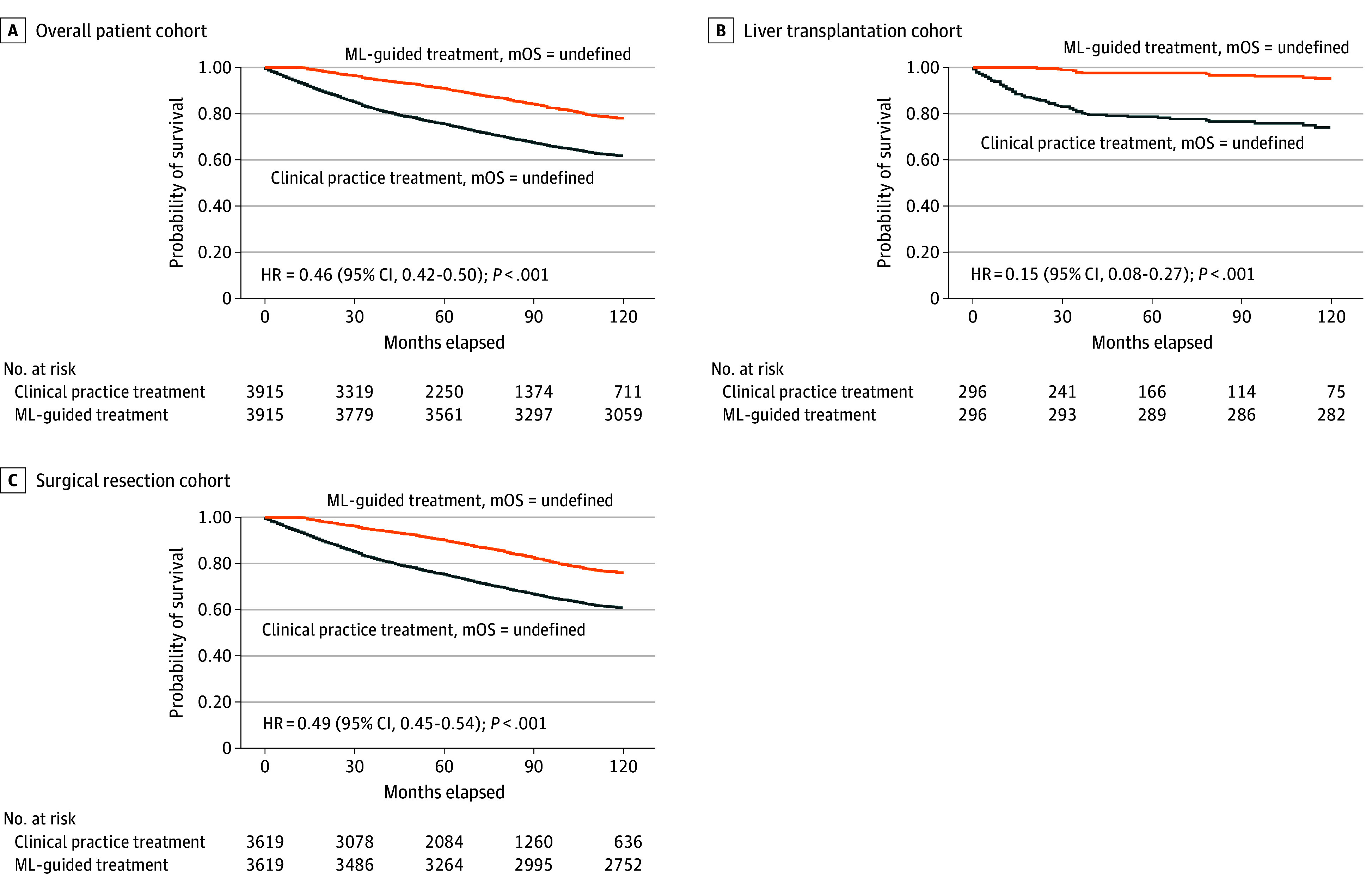
Counterfactual Survival Analysis Comparing Machine Learning (ML)–Guided vs Clinical Practice Treatments in the Derivation Cohort Kaplan-Meier survival curves comparing ML-guided and clinical practice treatments in the overall patient (n = 3915) (A), liver transplant (n = 296) (B), and surgical resection (n = 3619) (C) cohorts. HR indicates hazard ratio; mOS, median overall survival.

Counterfactual analysis estimated survival outcomes under ML-recommended vs actual treatment. In the overall cohort (n = 3915), ML-guided treatment was associated with improved survival (HR, 0.46 [95% CI, 0.42-0.50]; *P* < .001) (Figure 3A). Subgroup analyses confirmed consistent findings: in the LT group (n = 296), ML-guided treatment was also associated with superior outcomes (HR, 0.15 [95% CI, 0.08-0.27]; *P* < .001) (Figure 3B); and in the SR group (n = 3619), it was likewise favorable (HR, 0.49 [95% CI, 0.45-0.54]; *P* < .001) (Figure 3C).

### Validation With an External, Independent Cohort

In the external validation cohort, the models achieved an AUROC of 0.75 (95% CI, 0.70-0.80) for the LT cohort and 0.80 (95% CI, 0.75-0.85) for the SR cohort, suggesting that performance was maintained in the external validation (eTable 4 in Supplement 1). Risk stratification yielded similar results: in both LT and SR groups, patients categorized as low risk had significantly better OS than patients categorized as high risk (LT: HR, 0.24 [95% CI, 0.14-0.40]; *P* < .001; SR: HR, 0.32 [95% CI, 0.18-0.56]; *P* < .001) (eFigure 2A and B in Supplement 1). When stratified by donor type, the low-risk group showed superior survival (deceased donor liver transplant [DDLT]: HR, 0.07 [95% CI, 0.01-0.51]; *P* = .009; living donor liver transplant [LDLT]: HR, 0.28 [95% CI, 0.16-0.48]; *P* < .001) (eFigure 3A and B in Supplement 1). In the 4-group analysis, LT was associated with better survival in the LT low risk with SR high risk subgroup (HR, 0.29 [95% CI, 0.08-0.97]; *P* = .04), but was associated with worse survival in the LT high risk with SR low risk group (HR, 1.82 [95% CI, 1.01-3.28]; *P* = .05) (eFigure 4A-D in Supplement 1).

**Figure 4.**
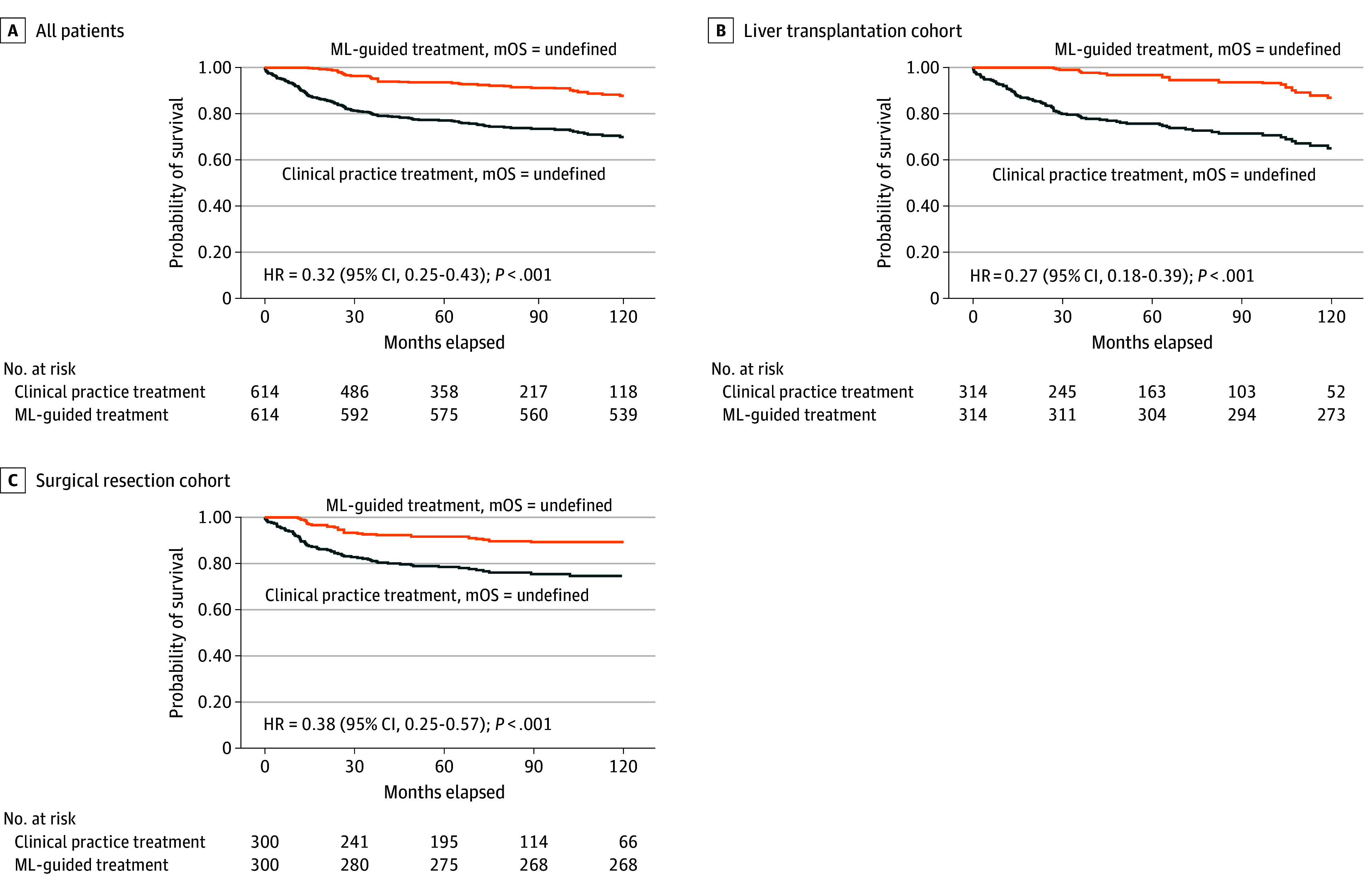
Counterfactual Survival Analysis Comparing Machine-Learning (ML)–Guided vs Clinical Practice Treatments in the External Cohort Kaplan-Meier survival curves comparing ML-guided and clinical practice treatments in the overall patient (n = 614) (A), liver transplant (n = 314) (B), and surgical resection (n = 300) (C) cohorts. HR, indicates hazard ratio; mOS, median overall survival.

Among 314 LT recipients, the model recommended SR for 243 (77.4%), including 8 DDLT and 235 LDLT cases. Among the 300 SR recipients, LT was recommended for 56 (18.7%). Notably, adherence to ML-based treatment recommendations resulted in significantly better survival outcomes compared with clinical practice decisions (Figure 4A; HR, 0.32 [95% CI, 0.25-0.43]; *P* < .001); within subgroups, the HR was 0.27 (95% CI, 0.18-0.39; *P* < .001) for LT recipients and 0.38 (95% CI, 0.25-0.57; *P* < .001) for SR recipients (Figure 4B and C). Simulated survival benefits of ML-guided treatment were also observed across donor types. Among DDLT recipients, ML-guided treatment was associated with improved estimated survival (HR, 0.35 [95% CI, 0.13-0.93]; *P* = .04). Similarly, among LDLT recipients, ML-guided treatment was associated with a statistically significant improvement in estimated survival (HR, 0.33 [95% CI, 0.23-0.47]; *P* < .001) (eFigure 5A and B in Supplement 1).

## Discussion

This cohort study developed and externally validated an ML-based decision-support model to optimize treatment selection between LT and SR for patients with HCC. While LT generally offers superior long-term survival, clinical decision-making remains complex due to limited organ availability and concerns about postresection hepatic functional reserve.^7,8,24^ The key contribution of this study is the potential identification of distinct patient subgroups—those deriving clear survival benefit from LT (LT-favorable) and those achieving comparable outcomes with SR (LT-nonfavorable)—through the integration of comprehensive clinical variables into ML algorithms

Current international guidelines, including those from the European Association for the Study of the Liver and the American Association for the Study of Liver Diseases, recommend treatment based on hepatic functional reserve, portal hypertension, and Milan criteria.^1,25^ SR is generally preferred for patients with Child-Pugh class A liver function and no significant portal hypertension, whereas LT is indicated for patients within Milan criteria.^1,25^ However, these frameworks often provide insufficient guidance in borderline cases, particularly when balancing risks such as donor shortages and tumor progression during transplant waitlisting.^26,27,28,29^ A comparison of major HCC guidelines reveals substantial variability in treatment recommendations for intermediate scenarios, underscoring the lack of consensus on selecting LT vs SR.^27^ Our study directly addresses these limitations by providing individualized, data-driven risk stratification beyond such conventional categories, offering more nuanced guidance for patients in the gray zone that current guidelines do not clearly inform.

Our model offers notable advantages over traditional statistical approaches by capturing complex, nonlinear associations among clinical variables.^17^ By incorporating 30 diverse clinical and tumor-related features, the model enabled a nuanced and individualized evaluation of patient risk that conventional methods cannot achieve. Indeed, studies have reported that ML-based prediction models can modestly outperform Cox regression in predicting posttransplant outcomes.^17^ Moreover, SHAP analysis further enhanced interpretability, identifying clinically relevant factors, tumor number, PIVKA-II, and creatinine, as major determinants in the LT cohort, and maximum tumor size and AFP in the SR cohort—consistent with established prognostic factors.^9,10^ The counterfactual analysis suggested that adherence to ML-guided treatment recommendations could improve survival compared with clinical practice decisions, highlighting the model’s potential to optimize individualized treatment allocation. These findings align with prior studies demonstrating the prognostic utility of ML algorithms in cancer treatment. For instance, previous work by members of our team validated an XGBoost-based model for Barcelona Clinic Liver Cancer stage C HCC,^30^ and similar ML-driven treatment optimization has shown clinical benefits in breast and lung cancers.^31,32^

Clinically, this model offers practical implications for optimizing organ allocation. In the internal cohort, the model recommended SR for 74.7% of patients who underwent LT and LT for only 19.4% of those who received SR. Similar findings were observed in the external validation cohort (77.4% and 18.7%, respectively). These results suggest that following the recommendations of the model may reduce overall LT volume while increasing its efficiency. In an LDLT-dominant area, this focused allocation may protect donor safety and enhance transplant ethics.^33^

Although our data were derived from an LDLT-dominant cohort, subgroup analyses demonstrated that the model also effectively stratified risk and esimated survival outcomes among DDLT recipients. These findings suggest that the prognostic capability of the model is not limited to LDLT settings, and in countries with DDLT-based systems and centralized allocation, ML model-based recommendations may have potential to complement existing frameworks by refining prioritization criteria to improve transplant utility and equity.^34^ Validation in larger DDLT cohorts is warranted to support such applications.

The clinical applicability of this model must be understood within the constraints of clinical practice. In LDLT-predominant settings, undergoing LT depends on the availability of a medically suitable and willing donor—a prerequisite that is neither guaranteed nor uniformly available. Accordingly, the model does not assume equal access to both treatments but instead provides individualized estimates of survival benefit under the hypothetical availability of either option. This framework may support the prioritization of transplant resources, refinement of institutional decision-making, and more informed discussions around optimal treatment strategies.

Finally, beyond optimizing treatment allocation, the model may serve as a communication tool. By providing individualized survival estimates under both LT and SR scenarios, clinicians may better inform patients of their expected prognosis, thereby enhancing shared decision-making and aligning treatment plans with patient values and expectations. However, its implementation in routine care would require attention to patient readiness, ethical concerns, and communication strategies.

### Limitations

This study has limitations. First, the retrospective design is inherently subject to selection bias and residual confounding, despite the use of a large nationwide registry, external validation, and counterfactual analysis. Therefore, findings should be interpreted with caution. Prospective validation—including randomized clinical trials involving patients with borderline indications for LT vs SR—is needed to confirm the safety and utility of ML-guided recommendations. Second, the model did not incorporate imaging biomarkers, genomic data, or longitudinal treatment response, which could further improve predictive accuracy. Future research should assess prospective performance and explore integration with radiomics and genomics to enhance decision-support in clinical workflows.

## Conclusions

In this cohort study of patients with HCC, ML enabled effective risk stratification and individualized treatment selection. These ML-guided decisions demonstrated the potential to improve survival in simulated analyses. These findings suggest that ML-informed strategies may enhance clinical outcomes and to optimize transplant resource allocation.

## Notes

Supplement 1.
eMethods.
**eTable 1.** Baseline Characteristics of the Derivation Cohort **eTable 2.** Baseline Characteristics of the External Cohort **eTable 3.** Performance of Machine Learning Algorithms in the Derivation Cohort **eTable 4.** Performance of Machine Learning Algorithms in the External Validation Cohort **eFigure 1.** Shapley Additive Explanation (SHAP) Summary Plots of Machine Learning (ML) Models **eFigure 2.** Kaplan-Meier Survival Analysis of Machine Learning–Defined Risk Groups in the External Cohort **eFigure 3.** Kaplan-Meier Survival Curves of Machine Learning–Defined Risk Groups Stratified by Donor Type in the External Validation Cohort **eFigure 4.** Combinatorial ML-Based Risk Stratification and Survival Analysis in the External Validation Cohort **eFigure 5.** Counterfactual Survival Analysis Comparing ML-Guided Versus Real-World Treatment Decisions Stratified by Donor Type in the External Validation Cohort

Supplement 2.
Data Sharing Statement
