# Supplementary material for: Machine Learning–Based Selection of Resection vs Transplant and Survival in Hepatocellular Carcinoma
Source: JAMA Netw Open. 2025 Sep 17;8(9):e2532353. doi: 10.1001/jamanetworkopen.2025.32353 (PMC12444571; doi:10.1001/jamanetworkopen.2025.32353)
Supplement: Supplement 1. — eMethods. eTable 1. Baseline Characteristics of the Derivation Cohort eTable 2. Baseline Characteristics of the External Cohort eTable 3. Performance of Machine Learning Algorithms in the Derivation Cohort eTable 4. Performance of Machine Learning Algorithms in the External Validation Cohort eFigure 1. Shapley Additive Explanation (SHAP) Summary Plots of Machine Learning (ML) Models eFigure 2. Kaplan-Meier Survival Analysis of Machine Learning–Defined Risk Groups in the External Cohort eFigure 3. Kaplan-Meier Survival Curves of Machine Learning–Defined Risk Groups Stratified by Donor Type in the External Validation Cohort eFigure 4. Combinatorial ML-Based Risk Stratification and Survival Analysis in the External Validation Cohort eFigure 5. Counterfactual Survival Analysis Comparing ML-Guided Versus Real-World Treatment Decisions Stratified by Donor Type in the External Validation Cohort [file jamanetwopen-e2532353-s001.pdf]

Supplementary Online Content

Kim HK, Han JW, Sung PS, et al. Machine learning–based selection of resection vs transplant and survival in hepatocellular carcinoma. JAMA Netw Open. 2025;8(9):e2532353. doi:10.1001/jamanetworkopen.2025.32353

eMethods.

eTable 1. Baseline Characteristics of the Derivation Cohort

eTable 2. Baseline Characteristics of the External Cohort

eTable 3. Performance of Machine Learning Algorithms in the Derivation Cohort

eTable 4. Performance of Machine Learning Algorithms in the External Validation Cohort

eFigure 1. Shapley Additive Explanation (SHAP) Summary Plots of Machine Learning (ML) Models

eFigure 2. Kaplan-Meier Survival Analysis of Machine Learning–Defined Risk Groups in the External Cohort

eFigure 3. Kaplan-Meier Survival Curves of Machine Learning–Defined Risk Groups Stratified by Donor Type in the External Validation Cohort

eFigure 4. Combinatorial ML-Based Risk Stratification and Survival Analysis in the External Validation Cohort

eFigure 5. Counterfactual Survival Analysis Comparing ML-Guided Versus Real-World Treatment Decisions Stratified by Donor Type in the External Validation Cohort

This supplementary material has been provided by the authors to give readers additional information about their work.

© 2025 Kim HK et al. *JAMA Network Open.*

eMethods.

Imputation of Missing Data

Missing values were imputed separately within the liver transplantation (LT) and surgical resection (SR) groups to preserve group-specific distributions. The derivation cohort included 296 patients in the LT group and 3,619 patients in the SR group. Missing numerical variables were imputed using a multivariate Iterative Imputer based on Bayesian ridge regression, which models each variable as a function of the others and iteratively fills in missing values. In the LT group, missingness was observed in total cholesterol (46 patients [15.6%]), PIVKA-II (39 [13.3%]), alpha-fetoprotein (AFP) (21 [7.0%]), sodium (10 [3.3%]), international normalized ratio (INR) (8 [2.6%]), platelet count (7 [2.4%]), creatinine (7 [2.4%]), albumin (7 [2.4%]), and bilirubin (6 [2.1%]). In the SR group, missing values were found in total cholesterol (565 [15.6%]), PIVKA-II (495 [13.7%]), AFP (254 [7.0%]), sodium (119 [3.3%]), INR (96 [2.7%]), platelet count (88 [2.4%]), creatinine (82 [2.3%]), albumin (82 [2.3%]), and bilirubin (77 [2.1%]). For categorical variables, probabilistic sampling based on observed distributions within each treatment group was applied. Hepatitis C virus infection was missing in 5 patients (1.7%) in the LT group and 53 (1.5%) in the SR group, while ascites was missing in 3 patients (1.0%) in the LT group and 33 (0.9%) in the SR group.

In the external validation cohort, which included 314 LT and 300 SR patients, missing data were minimal and observed only in the PIVKA-II variable: 14 patients (4.5%) in the LT group and 8 (2.7%) in the SR group. To ensure consistency, the same Iterative Imputer model that was trained on the derivation cohort was applied to the external data. No other variables had missing values in the external cohort.

© 2025 Kim HK et al. *JAMA Network Open.*

Counterfactual Survival Analysis

To assess the potential survival benefit of model-guided treatment selection, we performed a counterfactual survival analysis using a Cox proportional hazards framework with predicted mortality probability incorporated as a linear predictor. For each patient i, two risk probabilities were calculated using machine learning models:

$$p_i^{\text{actual}} = \text{predicted risk under actual treatment},$$

$$p_i^{\text{ML}} = \text{predicted risk under ML-recommended treatment}$$

A Cox proportional hazards model was fitted using p_i^{actual} as the covariate:

$$h_i(t) = h_0(t) \cdot \exp\left(\beta \cdot p_i^{\text{actual}}\right)$$

where $h_0(t)$ is the baseline hazard function and β is the estimated coefficient representing the effect of predicted mortality risk on the hazard. Using the estimated β and baseline cumulative hazard $H_0(t)$, the counterfactual linear predictor under the ML-recommended treatment was computed as:

$$\eta_i^{\text{ML}} = \beta \cdot p_i^{\text{ML}}$$

The counterfactual survival function was then calculated as:

$$\widehat{S_i^{\text{ML}}}(t) = \exp\left(-H_0(t) \cdot e^{\eta_i^{\text{ML}}}\right)$$

For each patient, the estimated survival time under the ML-guided treatment strategy was defined as the earliest time t such that $\widehat{S_i^{\text{ML}}}(t) \leq 0.5$. If no such time was found within the maximum follow-up of 120 months, the predicted survival time was censored at 120 months.

We constructed a combined dataset containing observed survival times under actual

© 2025 Kim HK et al. *JAMA Network Open.*

treatment and estimated survival times under ML-guided treatment. Kaplan-Meier curves were generated for both groups, and differences were assessed using log-rank tests and Cox regression. This framework enabled direct comparison between real-world and model-guided survival outcomes across the entire cohort, allowing estimation of the potential impact of individualized, ML-informed treatment decisions.

© 2025 Kim HK et al. *JAMA Network Open*.

eTable 1. Baseline Characteristics of the Derivation Cohort

	LT (n=296)	SR (n=3,619)	*P* value
Sex			.92
Male	236 (79.7%)	2,901 (80.2%)	
Female	60 (20.3%)	718 (19.8%)	
Age	54.0 (49.0 - 60.0)	58.0 (51.0 - 66.0)	<.001
Height, cm	167.4 (162.0 - 172.1)	166.3 (160.4 - 171.0)	.49
Weight, kg	68.9 (61.2 - 76.4)	65.9 (59.0 - 73.1)	<.001
Smoking (PY)	0.0 (0.0 - 15.0)	0.0 (0.0 - 20.0)	.06
Diabetes mellitus	64 (21.6%)	828 (22.9%)	.66
Hypertension	57 (19.3%)	1,340 (37.2%)	<.001
Total cholesterol, mg/dL	144.0 (110.0 - 168.5)	162.0 (139.0 - 186.0)	<.001
HBV	225 (76.5%)	2,504 (70.3%)	.03
HCV	23 (8.2%)	247 (7.3%)	.69
Alcohol	84 (28.5%)	1,042 (29.2%)	<.001
Cirrhosis	78 (26.4%)	699 (19.3%)	.005
Encephalopathy	20 (6.8%)	10 (0.3%)	<.001
Ascites	50 (19.9%)	153 (4.2%)	<.001
ECOG			<.001
0	139 (68.1%)	2,453 (86.3%)	
1	45 (22.1%)	347 (12.2%)	
2 or more	20 (9.9%)	44 (1.5%)	
Albumin, g/dL	3.4 (2.8 - 4.0)	4.2 (3.9 - 4.5)	<.001
Bilirubin, mg/dL	1.4 (0.9 - 2.5)	0.7 (0.5 - 1.0)	<.001
INR	1.2 (1.1 - 1.5)	1.1 (1.0 - 1.1)	<.001
Creatinine, mg/dL	0.9 (0.7 - 1.0)	0.9 (0.8 - 1.0)	.19
Sodium, mmol/L	139.0 (136.0 - 141.0)	140.0 (138.0 - 142.0)	<.001
ALT, U/L	36.0 (26.0 - 53.3)	31.0 (22.0 - 47.0)	.19
Platelet, 10^9/L	87.0 (59.5 - 126.5)	167.0 (130.0 - 212.0)	<.001
AFP, ng/mL	12.6 (4.7 - 97.4)	16.1 (4.3 - 208.2)	.07
PIVKA-II, mAU/mL	40.0 (20.0 - 195.5)	71.0 (26.4 - 485.0)	<.001
Tumor number	1.0 (1.0 - 2.0)	1.0 (1.0 - 1.0)	<.001
Maximum size	2.3 (1.5 - 3.6)	3.2 (2.2 - 5.0)	<.001
Invasion			
Portal vein	23 (7.8%)	212 (5.9%)	.23
Hepatic vein	1 (0.3%)	1 (0.0%)	.36
Bile duct	2 (0.7%)	47 (1.3%)	.51
Nodal metastasis	2 (0.7%)	49 (1.4%)	.47
Distant metastasis	2 (0.7%)	41 (1.1%)	.66

LT, liver transplantation; SR, surgical resection; HBV, Hepatitis B virus; HCV, Hepatitis C virus; ECOG, Eastern Cooperative Oncology Group; INR, International Normalized Ratio; ALT, alanine aminotransferase; AFP, alpha-fetoprotein; PIVKA, protein induced by vitamin K antagonist-II

© 2025 Kim HK et al. *JAMA Network Open.*

eTable 2. Baseline Characteristics of the External Cohort

	LT (n=314)	SR (n=300)	*P* Value
Sex			.12
Male	262 (83.4%)	235 (78.3%)	
Female	52 (16.6%)	65 (21.7%)	
Age	55.0 (51.0 - 60.0)	59.0 (52.0 - 66.0)	<.001
Height, cm	166.4 (161.3 - 170.1)	164.3 (160.5 - 169.2)	.23
Weight, kg	67.4 (61.5 - 73.2)	66.9 (58.8 - 72.9)	.13
Smoking (PY)	0.0 (0.0 - 0.0)	0.0 (0.0 - 0.0)	.01
Diabetes mellitus	77 (24.5%)	58 (19.3%)	.14
Hypertension	76 (24.2%)	81 (27.0%)	.46
Total cholesterol, mg/dL	147.0 (131.0 - 173.0)	160.0 (136.8 - 185.0)	<.001
HBV	186 (59.2%)	179 (59.7%)	.93
HCV	29 (9.2%)	11 (3.7%)	.005
Alcohol	92 (29.3%)	74 (24.7%)	.20
Cirrhosis	173 (55.1%)	34 (11.3%)	<.001
Encephalopathy	19 (6.1%)	2 (0.7%)	<.001
Ascites	81 (25.8%)	4 (1.3%)	<.001
ECOG			.004
0	294 (93.6%)	298 (99.3%)	
1	5 (1.6%)	0 (0.0%)	
2 or more	15 (4.8%)	2 (0.6%)	
Albumin, g/dL	3.2 (2.8 - 3.7)	4.2 (3.8 - 4.4)	<.001
Bilirubin, mg/dL	1.6 (0.7 - 7.1)	0.7 (0.5 - 0.9)	<.001
INR	1.3 (1.1 - 1.8)	1.1 (1.0 - 1.1)	<.001
Creatinine, mg/dL	140.0 (137.0 - 142.0)	141.0 (140.0 - 143.0)	.067
Sodium, mmol/L	31.0 (21.0 - 55.0)	29.5 (21.0 - 44.0)	<.001
ALT, U/L	71.5 (47.2 - 119.7)	167.5 (129.0 - 208.2)	.13
Platelet, 10^9/L	12.9 (4.0 - 99.6)	8.8 (3.2 - 136.5)	<.001
AFP, ng/mL	34.5 (14.0 - 137.7)	43.5 (20.0 - 206.0)	.16
PIVKA-II, mAU/mL	3.2 (2.8 - 3.7)	4.2 (3.9 - 4.4)	.01
Tumor number	2.0 (1.0 - 3.0)	1.0 (1.0 - 1.0)	<.001
Maximum size	2.6 (1.7 - 3.7)	3.0 (2.1 - 4.5)	<.001
Invasion			
Portal vein	53 (16.9%)	13 (4.3%)	<.001
Hepatic vein	7 (2.2%)	4 (1.3%)	.55
Bile duct	7 (2.2%)	4 (1.3%)	.55
Nodal metastasis	9 (2.9%)	4 (1.3%)	.26
Distant metastasis	3 (1.0%)	3 (1.0%)	>.99
Donor Type			
DDLT	28 (8.9%)		
LDLT	286 (91.1%)		

LT, liver transplantation; SR, surgical resection; HBV, Hepatitis B virus; HCV, Hepatitis C virus; ECOG, Eastern Cooperative Oncology Group; INR, International Normalized Ratio; ALT, alanine aminotransferase; AFP, alpha-fetoprotein; PIVKA, protein induced by vitamin K antagonist-II; DDLT, deceased donor liver transplantation; LDLT, living donor liver transplantation

© 2025 Kim HK et al. *JAMA Network Open.*

eTable 3. Performance of Machine Learning Algorithms in the Derivation Cohort

**LT**					
Model	Accuracy, 95% CI	Precision, 95% CI	Recall, 95% CI	F1 Score, 95% CI	AUROC, 95% CI
Logistic Regression	0.72 (0.67-0.77)	0.29 (0.24-0.34)	0.91 (0.88-0.94)	0.44 (0.38-0.50)	0.71 (0.66-0.76)
Random Forest	0.85 (0.81-0.89)	0.80 (0.75-0.85)	0.36 (0.31-0.41)	0.50 (0.44-0.56)	0.74 (0.69-0.79)
XGBoost	0.83 (0.79-0.87)	0.42 (0.36-0.48)	0.45 (0.39-0.51)	0.43 (0.37-0.49)	0.71 (0.66-0.76)
Support Vector Machine	0.80 (0.75-0.85)	0.47 (0.41-0.53)	0.82 (0.78-0.86)	0.60 (0.54-0.66)	0.82 (0.78-0.86)
LightGBM	0.85 (0.81-0.89)	0.44 (0.38-0.50)	0.64 (0.59-0.69)	0.52 (0.46-0.58)	0.77 (0.72-0.82)
CatBoost	0.82 (0.78-0.86)	0.67 (0.62-0.72)	0.36 (0.31-0.41)	0.47 (0.41-0.53)	0.71 (0.66-0.76)
**SR**					
Model	Accuracy, 95% CI	Precision, 95% CI	Recall, 95% CI	F1 Score, 95% CI	AUROC, 95% CI
Logistic Regression	0.76 (0.75-0.77)	0.36 (0.34-0.38)	0.73 (0.72-0.74)	0.48 (0.46-0.50)	0.76 (0.75-0.77)
Random Forest	0.84 (0.83-0.85)	0.42 (0.40-0.44)	0.59 (0.57-0.61)	0.49 (0.47-0.51)	0.77 (0.76-0.78)
XGBoost	0.80 (0.79-0.81)	0.38 (0.36-0.40)	0.68 (0.66-0.70)	0.49 (0.47-0.51)	0.77 (0.76-0.78)
Support Vector Machine	0.79 (0.78-0.80)	0.32 (0.30-0.34)	0.78 (0.77-0.79)	0.45 (0.43-0.47)	0.74 (0.73-0.75)
LightGBM	0.79 (0.78-0.80)	0.41 (0.39-0.43)	0.63 (0.61-0.65)	0.50 (0.48-0.52)	0.77 (0.76-0.78)
CatBoost	0.78 (0.77-0.79)	0.41 (0.39-0.43)	0.66 (0.64-0.68)	0.51 (0.49-0.53)	0.79 (0.78-0.80)

LT, liver transplantation; SR, surgical resection; AUROC, area under the receiver operating characteristic curve; CI, confidence interval

© 2025 Kim HK et al. *JAMA Network Open.*

eTable 4. Performance of Machine Learning Algorithms in the External Validation Cohort

**LT**				
Accuracy, 95% CI	Precision, 95% CI	Recall, 95% CI	F1 Score, 95% CI	AUROC, 95% CI
0.74 (0.69–0.79)	0.40 (0.34–0.46)	0.55 (0.49–0.61)	0.46 (0.40–0.52)	0.75 (0.70–0.80)

**SR**				
Accuracy, 95% CI	Precision, 95% CI	Recall, 95% CI	F1 Score, 95% CI	AUROC, 95% CI
0.77 (0.72–0.82)	0.42 (0.36–0.48)	0.60 (0.54–0.66)	0.50 (0.44–0.56)	0.80 (0.75–0.85)

LT, liver transplantation; SR, surgical resection; AUROC, area under the receiver operating characteristic curve; CI, confidence interval

© 2025 Kim HK et al. *JAMA Network Open*.

eFigure 1. Shapley Additive Explanation (SHAP) Summary Plots of Machine Learning (ML) Models

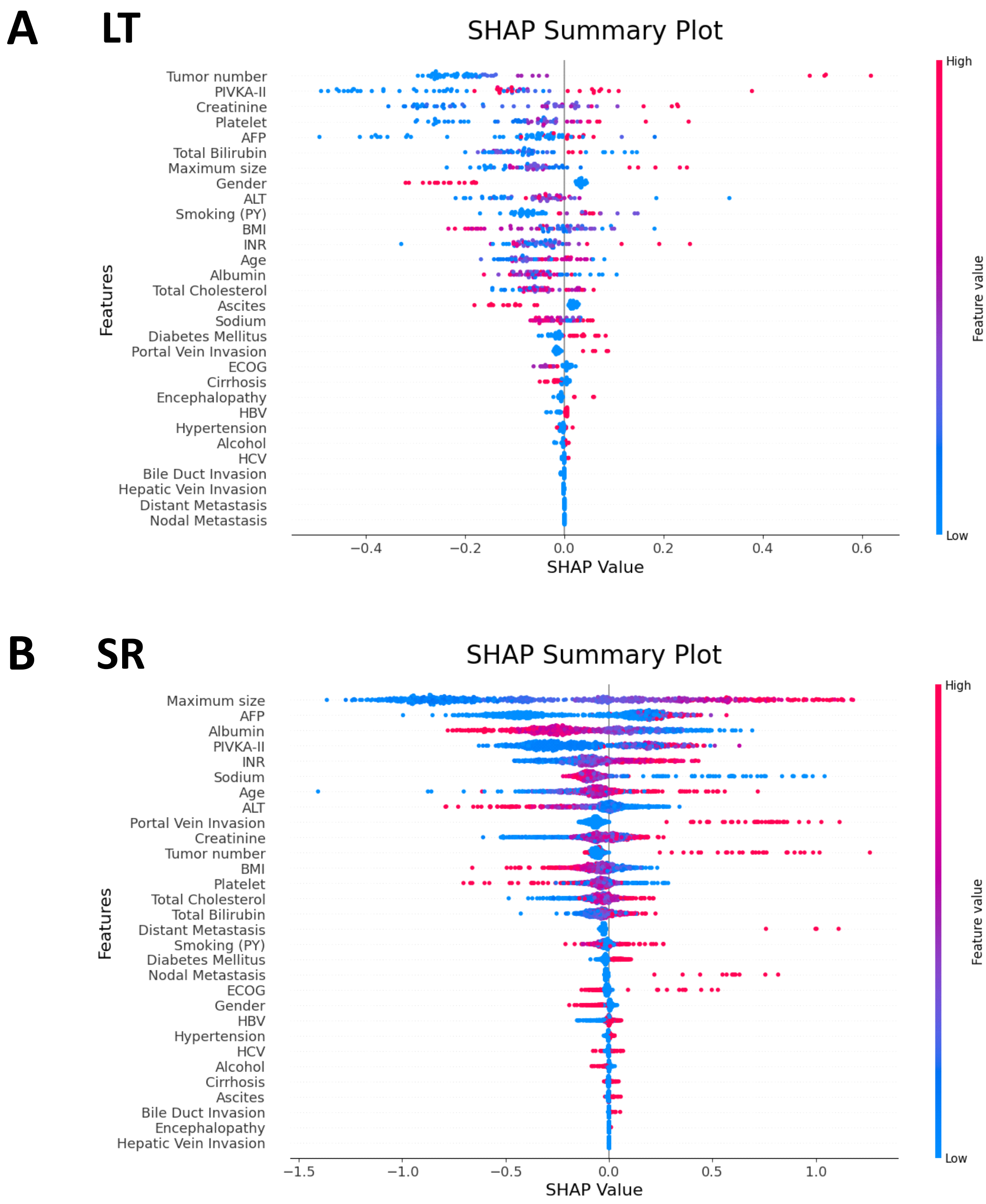

A, In the liver transplantation (LT) cohort, survival was predicted using the support vector machine model. B, In the surgical resection (SR) cohort, survival was predicted using the CatBoost model. Features are ranked based on their average SHAP value, indicating their relative impact on risk prediction. The color gradient represents feature values, with red indicating high values and blue indicating low values.

© 2025 Kim HK et al. *JAMA Network Open.*

eFigure 2. Kaplan-Meier Survival Analysis of Machine Learning–Defined Risk Groups in the External Cohort

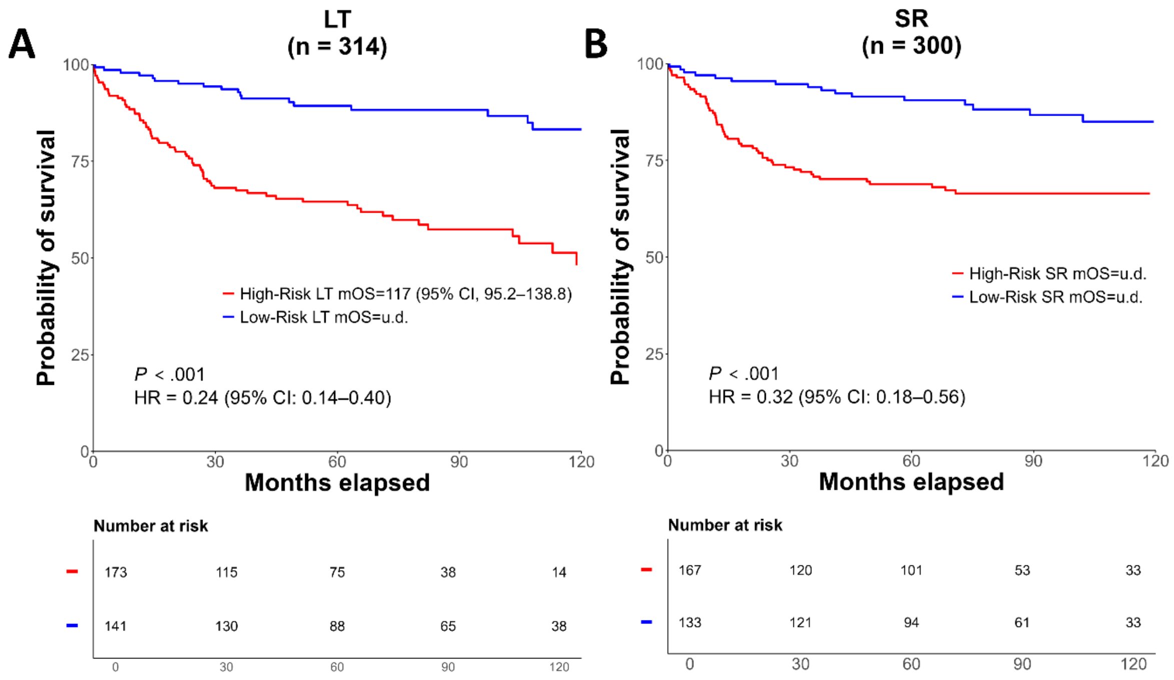

Patients were stratified into high- and low-risk groups using machine learning models for LT and SR. A, Kaplan-Meier survival curves comparing high-risk (n=173) and low-risk (n=141) groups within the LT cohort (n=314). B, Kaplan-Meier survival curves comparing high-risk (n=167) and low-risk (n=133) groups within the SR cohort (n=300). mOS, median overall survival; u.d., undefined; HR, hazard ratio; CI, confidence interval.

© 2025 Kim HK et al. *JAMA Network Open.*

eFigure 3. Kaplan-Meier Survival Curves of Machine Learning–Defined Risk Groups Stratified by Donor Type in the External Validation Cohort

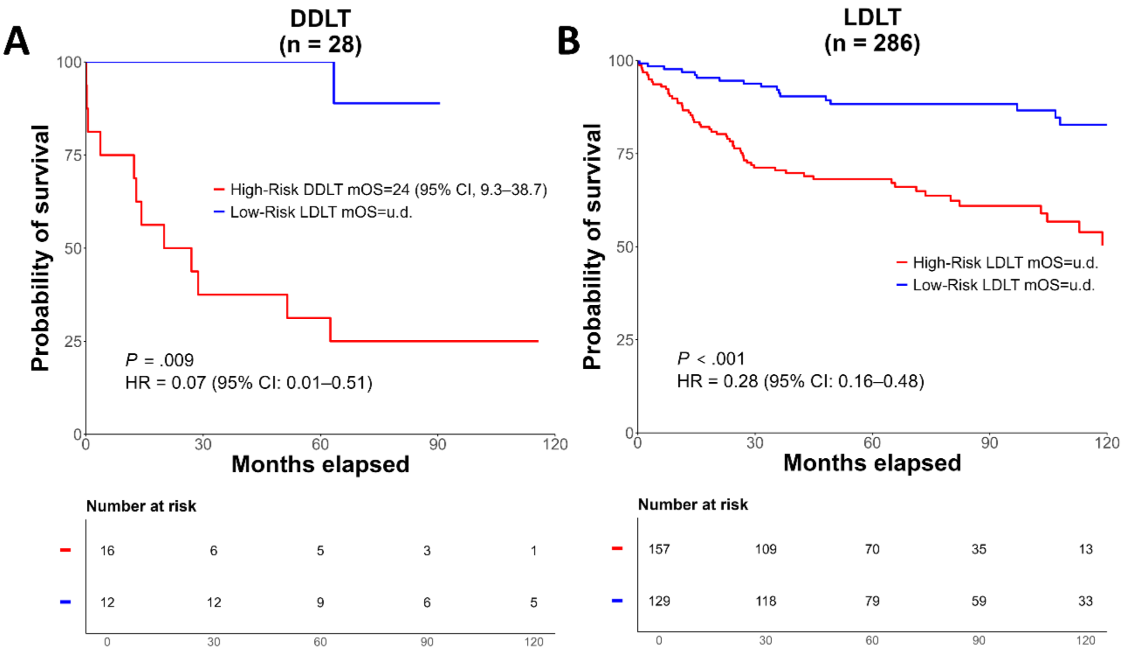

LT recipients in the external validation cohort were stratified into high- and low-risk groups using a machine learning model, and survival outcomes were compared separately for DDLT and LDLT subgroups. A, Kaplan-Meier survival curves comparing high-risk (n = 16) and low-risk (n = 12) groups among DDLT recipients (n = 28). B, Kaplan-Meier survival curves comparing high-risk (n = 157) and low-risk (n = 129) groups among LDLT recipients (n = 286). DDLT, deceased donor liver transplantation; LDLT, living donor liver transplantation; mOS, median overall survival; HR, hazard ratio; CI, confidence interval; u.d., undefined.

© 2025 Kim HK et al. *JAMA Network Open.*

eFigure 4. Combinatorial ML-Based Risk Stratification and Survival Analysis in the External Validation Cohort

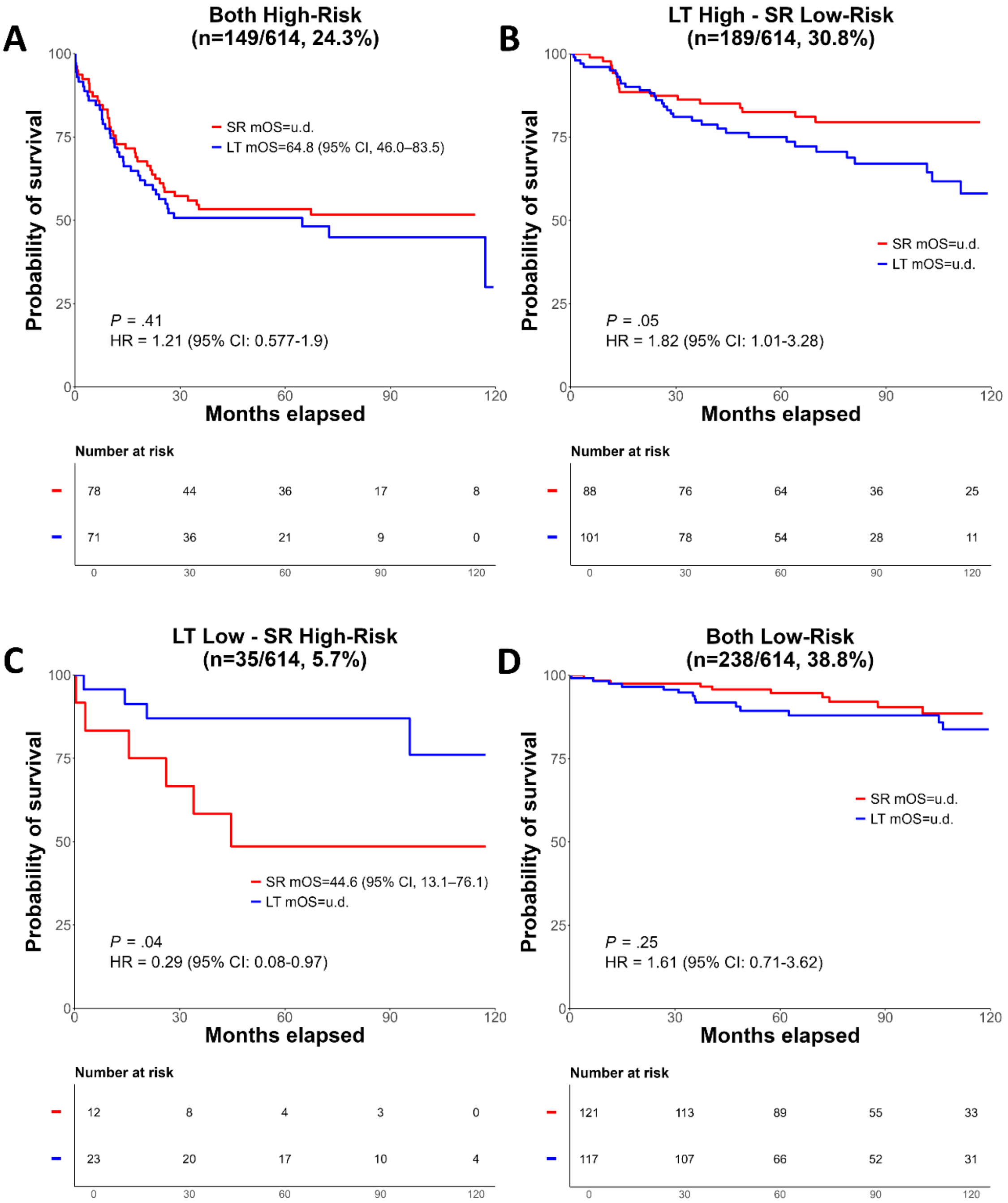

Patients in the external validation cohort (n=614) were stratified into four risk groups based on predicted survival outcomes for LT and SR. A, Kaplan-Meier survival curves comparing LT (n=71) and SR (n=78) in the Both High-Risk group (n=149). B, Kaplan-Meier survival curves comparing LT (n=101) and SR (n=88) in the LT High-Risk / SR Low-Risk group (n=189). C, Kaplan-Meier survival curves comparing LT (n=23) and SR (n=12) in the LT Low-Risk / SR High-Risk group (n=35). D, Kaplan-Meier survival curves comparing LT (n=117) and SR (n=121) in the Both Low-Risk group (n=238). mOS, median overall survival; u.d., undefined; HR, hazard ratio; CI, confidence interval.

© 2025 Kim HK et al. *JAMA Network Open.*

eFigure 5. Counterfactual Survival Analysis Comparing ML-Guided Versus Real-World Treatment Decisions Stratified by Donor Type in the External Validation Cohort

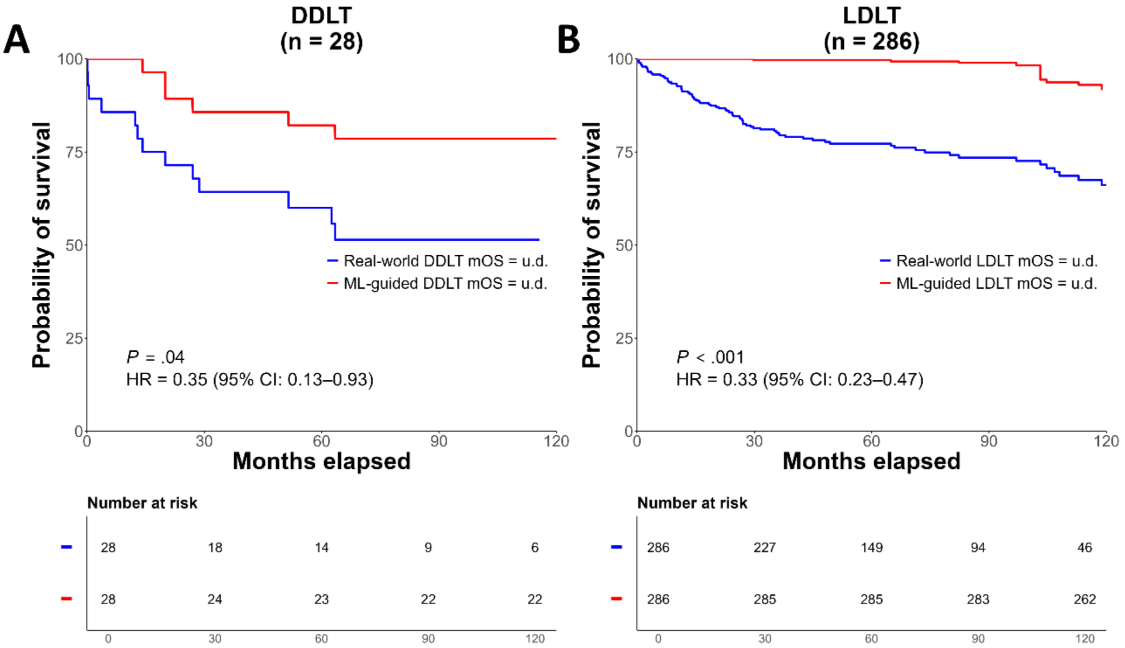

Counterfactual survival analysis comparing ML-guided versus real-world treatment decisions among DDLT and LDLT recipients in the external validation cohort. A, Kaplan-Meier survival curves comparing ML-guided versus real-world treatment decisions among DDLT recipients (n = 28). B, Kaplan-Meier survival curves comparing ML-guided versus real-world treatment decisions among LDLT recipients (n = 286). ML, machine learning; DDLT, deceased donor liver transplantation; LDLT, living donor liver transplantation; mOS, median overall survival; HR, hazard ratio; CI, confidence interval; u.d., undefined.

© 2025 Kim HK et al. *JAMA Network Open.*
