# Supplementary material for: Machine Learning–Based Selection of Resection vs Transplant and Survival in Hepatocellular Carcinoma
Source: JAMA Netw Open. 2025 Sep 17;8(9):e2532353. doi: 10.1001/jamanetworkopen.2025.32353 (PMC12444571; doi:10.1001/jamanetworkopen.2025.32353)
Supplement: Supplement 2. — Data Sharing Statement [file jamanetwopen-e2532353-s002.pdf]

Data Sharing Statement

Kim. Machine Learning–Based Selection of Resection vs Transplant and Survival in Hepatocellular Carcinoma. *JAMA Netw Open*. Published September 17, 2025. doi:10.1001/jamanetworkopen.2025.32353

Data
Data available: Yes
Data types: Deidentified participant data
How to access data: Deidentified individual participant data and a data dictionary will be made available upon reasonable request directed to the corresponding author (Dr. Ji Won Han, tmznjf@catholic.ac.kr), following institutional approval and execution of a data use agreement.
When available: With publication

Supporting Documents
Document types: Statistical/analytic code
How to access documents: All code used for model development, risk stratification, and statistical analysis will be publicly accessible at https://github.com/andrew8321/HCC_Machine-Learning.
When available: With publication

Additional Information
Who can access the data: researchers whose proposed use of the data has been approved
Types of analyses: for academic research only
Mechanisms of data availability: after approval of a proposal
